# Diffuse pruritic eruption and eosinophilia

**DOI:** 10.1016/j.jdcr.2024.04.004

**Published:** 2024-04-16

**Authors:** Serena X. Zhang, Kathleen E. Kramer, Vienna G. Katana

**Affiliations:** a Survival, Evasion, Resistance, and Escape (SERE), West Medical Department, Center for Security Forces Detachment North Island, San Diego, California; b Department of Dermatology, Naval Medical Center San Diego, San Diego, California

**Keywords:** eosinophilia, Hodgkin lymphoma, lichenoid dermatitis, paraneoplastic syndromes

## Case presentation

**Fig 1:**
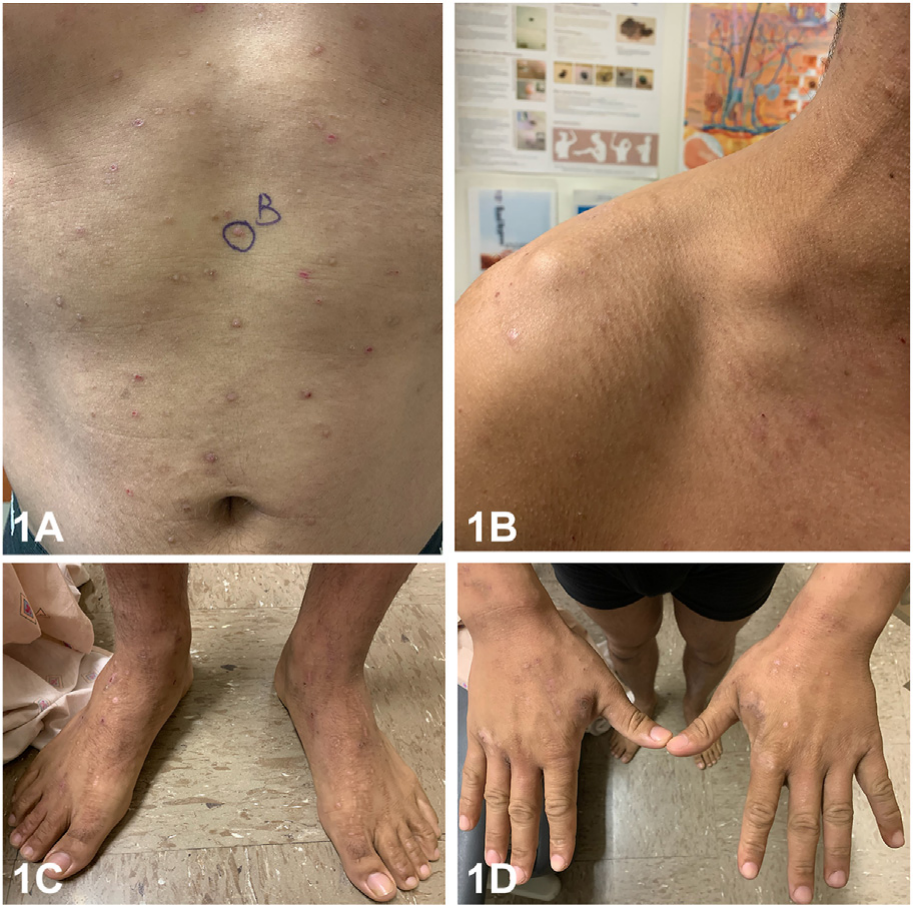

**Fig 2:**
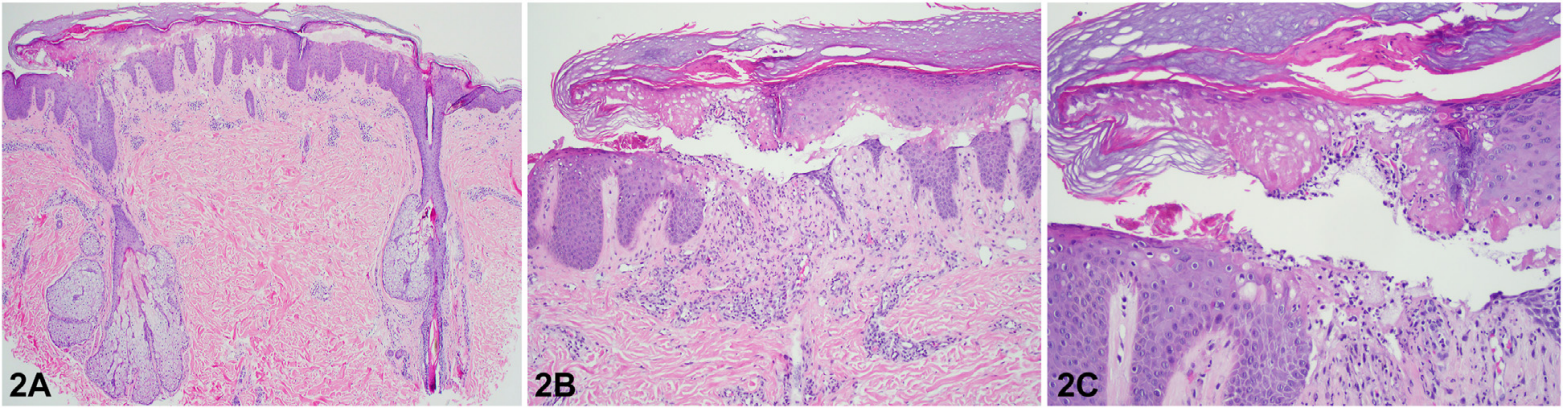

**Fig 3:**
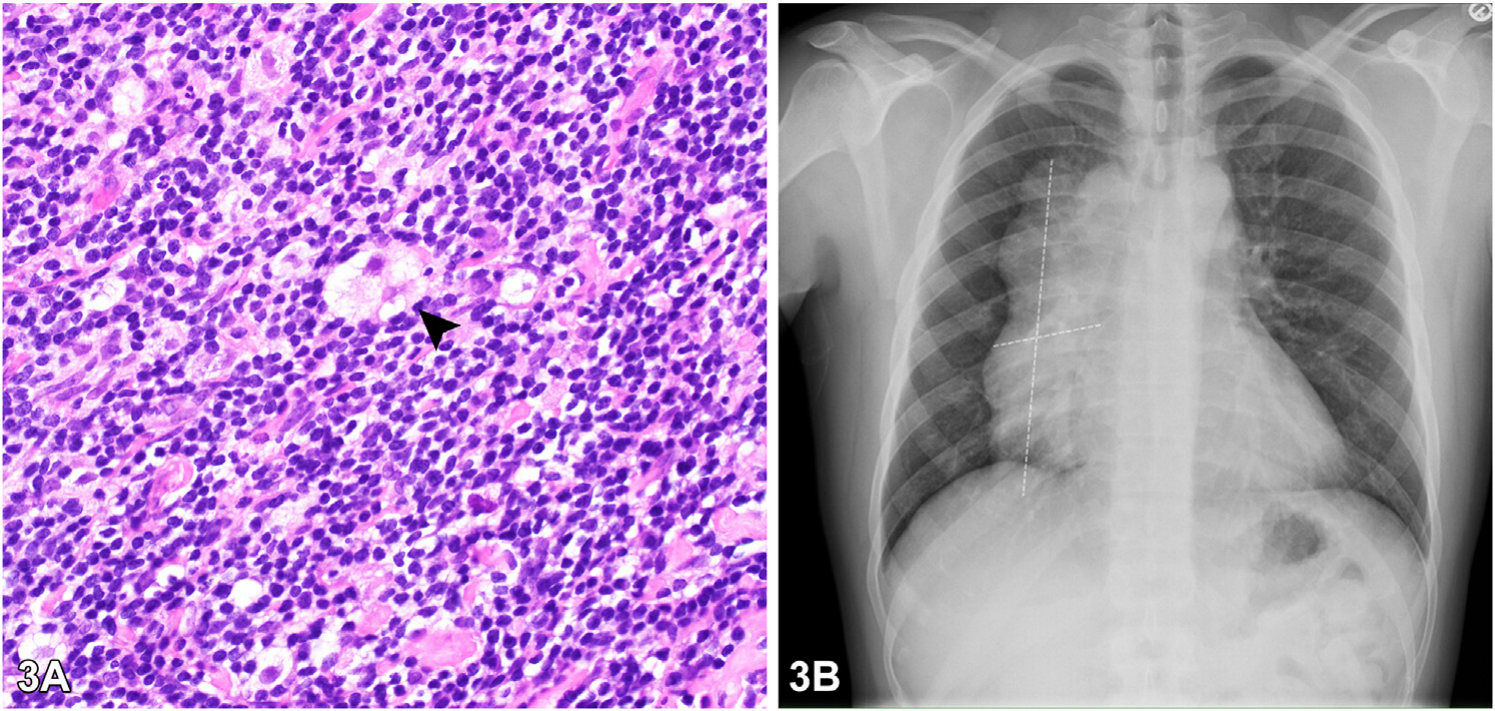

A 29-year-old male was referred for a 4-month history of a generalized pruritic rash. He reported a mild cough but denied other systemic symptoms, recent travel, exposures, and medication use. Exam showed excoriated flat topped violaceous papules in multiple stages of healing and a nontender right supraclavicular mass (Fig 1, *A*-*D*). Skin scrapings, mucosal and nail examination were negative. Skin biopsy was obtained (Fig 2, *A*-*C*) and direct immunofluorescence was negative. Bloodwork showed 19% eosinophils (absolute count of 2220/μL). Viral serology and anti-nuclear antibodies were normal. Chest x-ray and lymph node biopsy (Fig 3) were performed and confirmed the diagnosis. Treatment of the underlying cause resulted in the rapid resolution of the generalized rash and eosinophilia.

**Question 1: Based on histology, what is the most likely diagnosis?**

A. Parasitic infection
B. Lichen Planus
C. Erythema Multiforme
D. Cutaneous manifestation of a systemic disease
E. Subacute Cutaneous Lupus Erythematosus

**Answers:**

A. Parasitic infection – Incorrect. Patient’s history was negative for recent travel or exposure to parasitic diseases. Skin scrapings were negative for scabies mites. Arthropod bite reactions typically show eosinophilic spongiosis and wedge shaped superficial and deep mixed inflammatory infiltrate.
B. Lichen Planus – Incorrect. Although his exam demonstrated a lichenoid eruption, there were no oral or nail findings to suggest lichen planus. Histology was more suggestive of a lichenoid drug eruption, as eosinophils are not characteristically seen in classic lichen planus. He denied taking prescribed or over the counter medications.
C. Erythema Multiforme – Incorrect. Histology was reminiscent of Erythema multiforme with the acute interface changes and near full thickness necrosis, however the morphology and distribution of skin findings were disconcordant with EM. He had no oral mucosal lesions, acral targetoid papules, or plaques.
D. Cutaneous manifestation of a systemic disease – Correct. The skin eruption, nonspecific histology, supraclavicular mass, and eosinophilia were concerning for an underlying systemic disease.
E. Subacute Cutaneous Lupus Erythematosus – Incorrect. Subacute cutaneous lupus erythematosus is typically photodistributed and shows similar findings of acute interface dermatitis, however there was no basement membrane thickening or increased dermal mucin. Direct immunofluorescence and antinuclear antibodies were negative.

**Question 2: What is the most likely underlying systemic disease?**

A. Idiopathic hypereosinophilic syndrome
B. Hodgkin lymphoma
C. Kimura disease
D. Lung Adenocarcinoma
E. Sarcoidosis

**Answers:**

A. Idiopathic hypereosinophilic syndrome – Incorrect. Idiopathic hypereosinophilic syndrome is suspected when there is peripheral eosinophilia >1500 μL, with or without marked tissue eosinophilia, presence of end organ damage, and absence of another explanation.^1^ Although hypereosinophilic syndrome commonly affects the skin and can show eczematous and lichenoid changes,^1^ this case did not meet the criteria and the lymph node biopsy was concerning for malignancy.
B. Hodgkin lymphoma – Correct. This patient presented with a paraneoplastic lichenoid rash and new onset peripheral eosinophilia. Massive lymphadenopathy and Reed–Sternberg cells on lymph node biopsy support this diagnosis. Peripheral eosinophilia has been reported in 15% of Hodgkin lymphoma (HL) cases and may represent an early paraclinical sign.^2^ New onset eosinophilia especially within the first 3 months should raise the suspicion for HL.^2^
C. Kimura disease – Incorrect. Kimura disease can also present with massive lymphadenopathy and peripheral eosinophilia.^3^ Lymph node histopathology is required to rule out Kimura disease. Reed–Sternberg cells are not seen in this inflammatory disorder.
D. Lung Adenocarcinoma – Incorrect. Lung cancer is very common and accounts for more than 10% of all malignancy; however paraneoplastic eosinophilia is extremely rare in solid organ malignancies and should prompt a workup to exclude a secondary hematologic malignancy or bone marrow disease.^4^ Chest X-ray and advanced imaging did not show pulmonary involvement and Reed–Sternberg cells are not seen in the lymph nodes of metastatic lung adenocarcinoma.
E. Sarcoidosis – Incorrect. Sarcoidosis can mimic lymphoma when presenting with hilar/mediastinal lymphadenopathy, eosinophilia, and systemic symptoms.^3^ Histology did not show granulomatous dermatitis. Reed–Sternberg cells are not seen in sarcoidosis.

**Question 3: Which is true for cutaneous manifestations of this systemic disease?**

A. Cutaneous manifestations are the most common presenting finding
B. Cutaneous manifestations occur when the systemic disease directly involves the skin
C. Cutaneous manifestations are usually diagnosed after the systemic disease
D. Cutaneous manifestations persist after treatment of the systemic disease
E. Cutaneous manifestations that recur may signal relapse of the systemic disease

**Answers:**

A. Cutaneous manifestations are the most common presenting finding – Incorrect. Cutaneous paraneoplastic syndromes (PNS) are rarely the presenting clinical finding in HL patients (6.3% of cases); although if isolated pruritus is considered a PNS, this would be by far the most frequent presentation for this malignancy.^5^
B. Cutaneous manifestations occur when the systemic disease directly involves the skin – Incorrect. Cutaneous PNS are not related to direct tumor invasion nor distant metastasis to skin but rather malignancy induced immune dysfunction.^5^
C. Cutaneous manifestations are usually diagnosed after the systemic disease – Incorrect. Most patients with cutaneous PNS lack constitutional symptoms and are diagnosed simultaneously with HL.^5^ Dermatologist should be aware of HL associated cutaneous PNS where a timely diagnosis may improve prognosis.
D. Cutaneous manifestations persist after treatment of the systemic disease – Incorrect. Most PNS (such as pruritus and eczematous rashes) completely resolve with HL therapy.^5^ This patient had symptom resolution after 2 cycles of chemotherapy.
E. Cutaneous manifestations that recur may signal relapse of the systemic disease – Correct. Most PNS recur with HL relapse.^5^ Recurrence of cutaneous symptoms may serve as early detection of relapsed disease; therefore, these patients should have consistent and extended follow up with dermatology.

## Conflicts of interest

None disclosed.

## References

[1] Long C., Scott J.L., Flamm A. (2023). The dermatologic and histologic spectrum of hypereosinophilic syndrome. JAAD Case Rep.

[2] Jin J.J., Butterfield J.H., Weiler C.R. (2015). Hematologic malignancies identified in patients with hypereosinophilia and hypereosinophilic syndromes. J Allergy Clin Immunol Pract.

[3] García Carretero R., Romero Brugera M., Rebollo-Aparicio N., Vazquez-Gomez O. (2016). Eosinophilia and multiple lymphadenopathy: kimura disease, a rare, but benign condition. BMJ Case Rep.

[4] Abughanimeh O., Tahboub M., Abu Ghanimeh M. (2018). Metastatic lung adenocarcinoma presenting with hypereosinophilia. Cureus.

[5] El Fakih R., Bajuaifer Y.S., Shah A.Y. (2024). Paraneoplastic syndromes associated with classic Hodgkin lymphoma, a systematic literature review. Ann Hematol.

